# Differentially expressed tRFs in CD5 positive relapsed & refractory diffuse large B cell lymphoma and the bioinformatic analysis for their potential clinical use

**DOI:** 10.1186/s13062-019-0255-8

**Published:** 2019-11-27

**Authors:** Qingyuan Qu, Ying Li, Xiaosheng Fang, Lingyan Zhang, Chao Xue, Xueling Ge, Xin Wang, Yujie Jiang

**Affiliations:** Department of Hematology, Shandong Provincial Hospital Affiliated to Shandong University, No.324, Jingwu Road, Jinan, Shandong 250021 People’s Republic of China

**Keywords:** tRFs, DLBCL, Bioinformatics, Hub gene

## Abstract

**Background:**

Patients diagnosed as diffuse large B cell lymphoma (DLBCL) with CD5 positive normally have a worse outcome and poorly respond to the regulatory treatment strategy.

**Results:**

We recently reported differently expressed tRFs and their potential target-genes of tRFs in patients with CD5+ R/R DLBCL. Differently expressed tRFs were detected by Illumina NextSeq instrument and the results were verified by quantitative real-time reverse transcription-PCR. tRF2Cancer database was searched to compared with the results. Further research was performed through bio-informatic analysis including gene ontology (GO) and pathway enrichment analyses, etc. A total of 308 tRFs were identified. Two sequences (AS-tDR-008946, AS-tDR-013492) were chosen for further investigated.

**Conclusions:**

The results of Bioinformatics analysis revealed that the target genes including NEDD4L and UBA52 and several associated pathways including PI3K/AKT and MAPK/ERK might be involved in the development of CD5+ R/R DLBCL. Our preliminary study on the associated tRFs might provide a valuable measure to explore the pathogenesis and progression of CD5+ R/R DLBCL.

**Reviewers:**

This article was reviewed by Zhen Qing Ye, Nagarajan Raju and Jin Zhuang Dou.

## Background

Diffuse large B-cell lymphoma (DLBCL) is one of the most common lymphomas in adults which accounting for 38% of non-Hodgkin’s lymphoma (NHL) in China [1]. The combination of the anti-CD20 monoclonal antibody (rituximab) and cyclophosphamide, doxorubicin, vincristine, prednisone (R-CHOP) is the standard treatment for most of the new-diagnosed patients with DLBCL. However, a large subset of patients (30–40%) with DLBCL with high-risk factors often have a poor prognosis even though they received intensive combine chemotherapy or bio-target treatment [2]. A sub-group of DLBCL patients with CD5 positive exhibit more aggressive course of the disease and a considerable number of them will develop into relapsed & refractory (R/R) DLBCL. Furthermore, patients with CD5+ DLBCL in Asian countries showed a female predominance [3]. The exact mechanism of the pathogenesis, progression, and chemotherapy-resistance of these patients is still unknown and it may be related to multiple oncogene mutations and abnormal signal pathways. Therefore, investigating the accurate molecular mechanisms of the pathogenesis and progression of CD5+ R/R DLBCL is extremely meaningful.

It has been confirmed that small non-coding RNAs play important roles in the classification, diagnosis, treatment selection, and prognosis of DLBCL [4–6]. The tRFs, which derived from tRNA cleavage in a specific manner, are groups of abundant non-coding RNAs only less than miRNAs and were proved can play roles similar to miRNA in some biological processes [7]. Different from miRNA, tRFs are highly conserved, tissue-specific and time-specific and are reported to express stability in almost all types of cells and organisms and can be detected in body fluid [8, 9]. Compared with miRNAs, tRFs have their advantages to be used as candidate fluid-based biomarkers. It has been reported that tRFs participate in many biological processes including cell proliferation, viral reverse transcriptase activation, gene expression, RNA procession regulation, DNA damage response modulation, and tumor suppression [10]. The former study had proved that a tRF sequence is down-regulated in B cell lymphoma and can modulate proliferation and the DNA damage response [11]. Therefore, we deduce that tRFs might modulate the pathogenesis and infiltration of malignant B cells through their target genes. In this study, we aimed to investigate the profiles of differently expressed tRFs between CD5+ R/R DLBCL and healthy individuals. The most significantly expressed tRFs and their potential target genes will also be discussed by bio-informatic analysis in our study.

## Methods

### Patients

Three female patients were enrolled in this study at Shandong Provincial Hospital (Shandong, China). They were diagnosed as typical R/R DLBCL with CD5+ according to the entries formulated by ESMO Clinical Practice Guideline [12]. We collected the peripheral venous blood of them on the second day of hospitalization every time and stored the samples in -80^o^ fridge. In the end, three samples were chosen to analyze after the patient was confirmed as progressive disease or no response to the second/third line of chemotherapy. The result of Fluorescent in-situ hybridization (FISH) analysis from the biopsy of each patient showed that the rearrangement of BCL2, BCL6, MYC, and P53 were all negative. All three patients received a re-biopsy when they were diagnosed as R/R DLBCL and the pathological results were confirmed to be DLBCL. Clinical and laboratory data of the patients were extracted from medical records (Table 1). All three patients received at least three cycles of R-CHOP regimen before the disease progression or relapse. Three female healthy adults were enrolled as the control group. Their ages are 42, 55, and 61, respectively. All of the control samples were collected from the physical examination center of Shandong Provincial Hospital. The peripheral venous blood was collected from each participant through venipuncture and conserved in Ethylenediaminetetraacetic acid (EDTA) containing tubes for RNA extraction. Relevant ethical permission was obtained for the use of all samples. At the end of the follow-up, all three patients died of disease progression. This study was conducted in accordance with the Helsinki Declaration of 1975 which was revised in 2008 and approved by the research institutions and hospitals human research ethics committees. Informed consent was obtained from all participants and the confidentiality of data was assured for them.

**Table 1:** Clinical & Laboratory Characteristics of Patients

Parameter	Case 1	Case 2	Case 3
Age (years)	38	65	62
Sex	Female	Female	Female
IHC lymphoma subtype	GCB	GCB	Non-GCB
Phenotypes	CD5(+), CD10(+), Bcl-2(+), Bcl-6(+), CD20(+), CD3(+), MUM-1(−), CD7(+), CycinD1(−)	CD5(+), CD10(−), Bcl-2(+), Bcl-6(+), CD20(+), CD3(+), MUM-1(+), PAX-5(+), CD68(−), CycinD1(−)	CD5(+), CD10(−), Bcl-6(+), CD20(+), CD3(−), MUM-1(+), CD56(−), CycinD1(−)
B symptoms	Y	Y	N
Fever	Y	Y	N
Night sweats	Y	N	N
Weight loss	Y	N	N
Bulky disease (> 7.5 cm)	N	N	N
> 2 extranodal sites	Y (terminal ileum, stomach, liver)	Y (colon, chest wall)	Y (neck region, nasopharynx)
LDH (120–250 U/L)	5233	245.5	267.9
β2-microglobulin (1.0–3.0 mg/L)	3.4	3.39	1.5
Ki-67	70%	80%	50%
Stage	IV	IV	III
R-IPI prognostic group	5	3	4
Bone marrow involvement	N	N	N
Complications	Malnutrition sinus Tachycardia	Pulmonary infection Cholecystitis Hypertension	T2DM
Treatment	R-CHOPx3(PR) → R-CHOPx1 (PD) → R-Gemoxx2(NR)	R-CHOPx4(PR) → R-CHOPx3(SD) → R-Gemoxx4(PR) → R-IMVP16(NR)	R-CHOPx3(NR) → DICE (NR) → R-DHAPx2(PD)

*LDH* Lactate dehydrogenase T2DM: Type 2 diabates

*R-CHOP* rituximab plus cyclophosphamide, pharmorubicin, vincristine Sulfate (Oncovin), and prednisone

*R-Gemox* rituximab plus gemcitabine and oxaliplatin

*R-IMVP16* rituximab plus ifosfamide, methotrexate, and etoposide

*DICE* dexamethasone, ifosfamide, cisplatin, and etoposide

*R-DHAP* rituximab plus dexamethasone, high-dose cytarabine, and cisplatin (platinum)

*Case 2* R-IMVP16 regimen was chosen for the patient due to her poor physical state

*Case 3* After three courses of R-CHOP, DICE was given to the patient without rituximab because the patient refused to use it

### Total RNA isolation and RNA sequencing

Total RNA from patients’ peripheral blood was isolated by TRIzol (Invitrogen), RNA integrity was monitored by electrophoresis in denaturing polyacrylamide gels from Nanodrop. A commercial kit for tRF & tiRNA-seq library preparation was used in this study which includes 3′-adapter and 5′-adapter ligation adaptor ligation, cDNA synthesis, and library PCR amplification. The prepared tRF & tiRNA-seq libraries are finally quantified using Agilent BioAnalyzer 2100, then sequenced using Illumina NextSeq 500. For standard small RNA sequencing on Illumina NextSeq instrument, the sequencing type is 51-bp single-read at 10 M reads.

### Data analysis of tRFs & tiRNAs

Sequencing quality was examined by FastQC software. After Illumina quality control, the sequencing reads were 5′, 3′-adaptor trimmed, filtered for ≥15 nt by cutadapt software, and aligned to mature-tRNA and pre-tRNA sequences from GtRNAdb using NovoAlign software (v2.07.11). The remaining reads are aligned to the transcriptome sequences (mRNA /rRNA /snRNA /snoRNA /piRNA /miRNA). The expression profiling and differential expression of tRFs & tiRNAs & known miRNAs were calculated based on normalized TPM. When comparing two groups of profile differences (such as disease vs control) use the normalized tag number of tRNAs annotated in GtRNAdb, including the tag count of each sample, the “fold change” (i.e. the ratio of the group averages) between the groups for each tRF/tiRNA is computed. The statistical significance of the difference may be conveniently estimated by t-test. Microsoft Excel’s Data/Sort & Filter functionalities were used to filter the analysis outputs and rank the differentially expressed genes according to fold change, *p*-value, etc. The volcano plot and scatter plots were performed based on “Test *vs* Control” tRFs & tiRNAs with TPM values [13]. The data were z-score standardized, then hierarchical clustering was performed using the genes whose quantile probabilities of coefficient variation (CV) are within 0.5 and 0.85 based on TPM counts of the samples in one comparison (Test vs Control). Graphics were performed with R package.

### Quantitative RT-PCR analysis

Total RNA was extracted from blood with the use of TRI REAGENT B (MRCGENE) and their quantity and quality were determined using NanoDrop® ND-1000. Five μg RNA was reverse transcribed with the use of Reverse Transcription Primer. Some pretreatments was performed before library preparation including 3′-aminoacyl deacylation to 3′-OH for 3′ adaptor ligation, 3′-cP (2′,3′-cyclic phosphate) removal to 3′-OH for 3′ adaptor ligation, 5′-OH phosphorylation to 5′-P for 5′-adaptor ligation, m1A and m3C demethylation. Quantitative RT-PCR was carried out with the use of the ViiA 7 Real-time PCR System (Applied Biosystems). Primers for amplification of sequences were indicated in Table 2. Reactions were incubated for 10 min at 95 °C followed by 40 cycles for 10 s at 95 °C and 1 min at 60 °C. All reactions were run in triplicate and relative expression was analyzed by means of the comparative cycle threshold method (2^-ΔΔCt^) according to the manufacturer (Applied Biosystems). Values were expressed as fold change and compared with the control group.

**Table 2:** Primers for Amplification of Sequences

	Bidirectional primer sequences	Annealing temperature (°C)	Length of the product (bp)
U6 (reference gene)	F:5’GCTTCGGCAGCACATATACTAAAAT3’ R:5’CGCTTCACGAATTTGCGTGTCAT3’	60	89
AS-tDR-000045	F:5′ GTCCGACGATCGTCAGGATG3’ R:5′ GTGTGCTCTTCCGATCTGCTC3’	60	44
AS-tDR-008640	F:5′ TACAGTCCGACGATCGAAGC3’ R:5′ TGTGCTCTTCCGATCTGAAATAA3’	60	51
AS-tDR-008946	F:5′ CTACAGTCCGACGATCTCCG3’ R:5′ CTCTTCCGATCTCGGTCCTT3’	60	45
AS-tDR-011395	F:5′ GTTAACCGAAAGGTTGGTGGTA3’ R:5′ CCGATCTACTCAGTAATGGTAACG3’	60	49
AS-tDR-011461	F:5′ CTACAGTCCGACGATCAGGG3’ R:5′ TGCTCTTCCGATCTACGAGAAT3’	60	47
AS-tDR-013487	F:5′ ATCGCTAAGGAAGTCCTGTGC3’ R:5′ TGTGCTCTTCCGATCTGCTG3’	60	42
AS-tDR-013492	F:5′ AGTCCGACGATCCTGTCACG3’ R:5′ GTGTGCTCTTCCGATCTTGTCT3’	60	46
AS-tDR-007217	F:5′ AGTCCGACGATCGCATTGTG3’ R:5′ CTCTTCCGATCTGGCGAGAAT3’	60	54
AS-tDR-003278	F:5′ ACAGTCCGACGATCTAGAATTCTC3’ R:5′ CTCTTCCGATCTCGTGGCAG3’	60	46

### Bioinformatics analysis

A database on tRFs, tRFs2Cancer (http://rna.sysu.edu.cn/tRFfinder/predict.php, version 1.0), was utilized to analyze the similarity and dissimilarity of homologous tRNA fragment. The tRF sequences were loaded to a homemade software based on Targetscan and Miranda to predict the potential genes. The predicted targets for each tRF were loaded to the DAVID database [14] and ClueGO [15] in order to perform gene ontology (GO) annotation and pathways search including biological process (BP), cellular component (CC), molecular function (MF) and KEGG (Kyoto Encyclopedia of Genes and Genomes) [16]. The *Homo sapiens* protein-protein interaction (PPI) network used in this analysis was retrieved from the STRING database (highest confidence: 0.900) [17]. The PPI network was constructed with the target genes of the two sequences separately, and core genes were visualized using Cytoscape 3.6.1 [18]. MCC, DMNC, betweenness, and stress algorithms were used to identify the hub genes and produce the output using cytoHubba [19]. Hub genes of PPI networks are highly connected nodes with particular biological properties.

## Results

### Overview of the tRFs & tiRNAs profiling

**Fig. 1:**
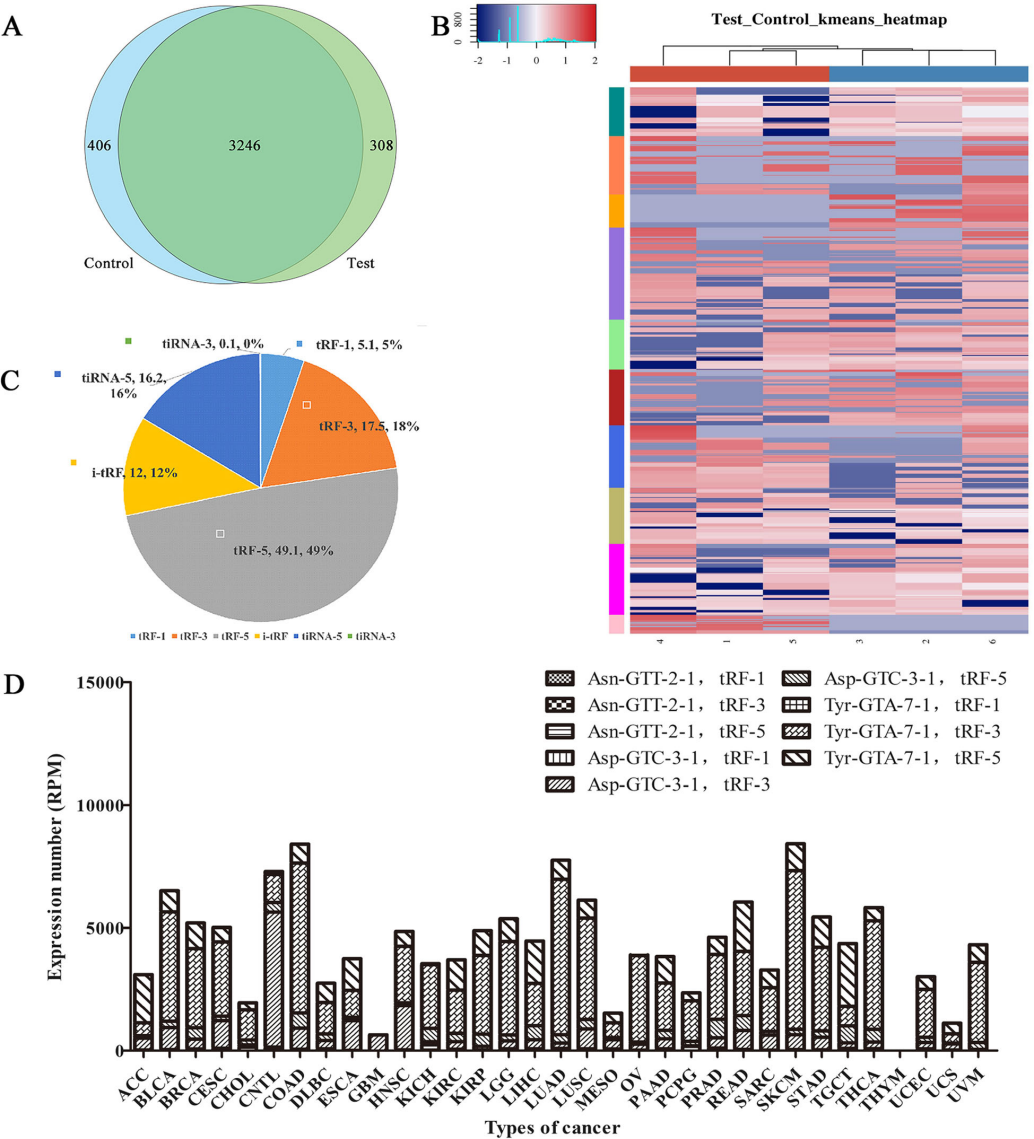
Overview of the tRFs & tiRNAs profiling and Data from tRF2 Cancer. **a** Venn diagram of the number of genes. The plot shows the number of genes that both expressed in common and expressed specifically. **b** Hierarchical cluster analysis of differential expression genes. The color in the panel represents the relative expression level (log2-transformed). Blue represents an expression level below the mean and red represents an expression level above the mean. The colored bar top at the panel showed the samples group, red indicates the experimental group and blue indicates the control group. Each row represents one gene and all the selected genes were categorized into no more than 10 clusters based on K-means clustering, each column represents one sample. The colored bar at the right side of the panel indicated the divisions which were performed using K-means. **c** Pie plot for all tRFs & tiRNAs of each group using all unique expressed tRFs & tiRNAs. The values in the pie plot are the type of tRFs & tiRNAs and the expression level (TPM values) percentage of each subtype of tRFs or tiRNAs, respectively. **d** Distribution of tRFs expression across cancers. DLBC stands for diffuse large B-cell lymphoma (DLBC)

To determine the differently expressed tRFs & tiRNAs profiles in CD5+ R/R DLBCL, we recruited three cases and three healthy controls. Their blood RNAs were sequenced with RNA-sequencing technology. (GSE140225 ) after quality control filtering of the raw sequencing reads, 12,106,086, 11,250,357 and 11,481,802 clean reads were generated for three CD5+ R/R DLBCL patients, and 7,815,334, 9,634,464 and 9,365,532 clean reads were generated for three healthy controls. Totally, 308 tRFs & tiRNAs were identified to expressed specificly in patients with CD5+ R/R DLBCL and 406 tRFs & tiRNAs were identified to expressed specificly in the control group. Venn diagram of the RNA-sequencing data was shown in Fig. 1a. The classification of the 308 and 406 tRFs & tiRNAs was shown in Additional file 1: Figure S1A, 1B. The Scatter-Plot is performed to provide a visualization method for assessing the tRF & tiRNA expression variation (or reproducibility) between the two compared groups of samples (Additional file 1: Figure S2A). We also performed the volcano plot to provide quick visual identification of the tRFs & tiRNAs displaying large-magnitude changes (Additional file 1: Figure S2B).

The hierarchical cluster analysis showed that the expression pattern of the largest coefficient of variation tRFs & tiRNAs. After the data was z-score standardized, the hierarchical clustering was performed using the genes whose quantile probabilities of coefficient variation (CV) are within 0.5 and 0.85 based on TPM counts of the samples in one comparison (Test vs Control). Each row represents one gene and all selected genes were categorized into no more than 10 clusters based on K-means clustering, each column represents one sample. The result from Hierarchical Clustering shows a distinguishable tRF & tiRNA expression profiling among samples (Fig. 1b).

Among subtypes of tRFs & tiRNAs, tRF-5 has the highest expression level which accounts 49% and the lowest part is tiRNA-3. As is shown in Fig. 1c, the pie plot is used to show the unique tRFs & tiRNAs of the expressed level percentage of each sub-type.

### Data from tRF2Cancer

tRF2Cancer is the first web server for identifying tRFs and their expression in cancers from small RNA deep-sequencing data. By 2018, there are 33 Cancer types in it, including DLBCL. Although the sample number of DLBCL is less than many other types of cancer in this database, we can still find out some tendencies. Four types of tRFs were highly expressed in our DLBCL simples, with Tyr-GTA-7-1, tRF-3 being the highest expressed, followed by Tyr-GTA-7-1, tRF-5, Asp-GTC-3-1, tRF-3, Asp-GTC-3-1, tRF-5. Above all, tRF-3 and tRF-5 are the highly expressed tRFs types in DLBCL. Distribution of tRFs expression across different types of cancers was shown in Fig. 1d.

### Further selection for tRF sequences

When comparing the two groups of profile differences, we use the normalized tag number of tRNAs annotated in GtRNAdb, including the tag count of each sample, the fold change between the groups for each tRF& tiRNA is computed. The statistical significance of the difference was estimated by t-test. The tRFs&tiRNAs which fold changes ≥2 and *p*-values ≤0.05 are selected as the significantly differentially expressed tRFs & tiRNAs. Four sequences were significantly upregulated and six were significantly downregulated in patients with CD5+ R/R DLBCL. Then according to the tag count of the reading sequence of each sample, we further narrowed the scope of research. Sequences only expressed in the control group or experimental group were chosen, including three upregulated sequences (AS-tDR-000045, AS-tDR-008946, AS-tDR-013492) and four downregulated sequences (AS-tDR-011383, AS-tDR-013475, AS-tDR-013487, AS-tDR-011395). To confirm the content of the differentially expressed tRFs in the patient’s group, we performed quantitative real-time PCR. Among the seven sequences, four sequences (AS-tDR-000045, AS-tDR-011383, AS-tDR-013475, AS-tDR-013487) were excluded because there are no significant differences between the experimental group and te control group. Finally, we verified the three tRFs including AS-tDR-011395, AS-tDR-008946, and AS-tDR-013492. The results of RT-PCR were consistent with the sequencing analysis. The characteristics of these sequences are shown in Table 3. And the structure of AS-tDR-013492 and AS-tDR-008946 is shown in Additional file 1: Figure S2C.

**Table 3:** The characteristics of three sequences assured by quantitative PCR

tDRs ID	tRNA	type
Up-regulated		
AS-tDR-013492	Asp-GTC-3-1	i-tRF
AS-tDR-013492	Asp-GTC-5-1	i-tRF
AS-tDR-008946	Tyr-GTA-7-1	tRF-3
Down-regulated		
AS-tDR-011395	Asn-GTT-2-1	tRF-3
AS-tDR-011395	Asn-GTT-2-2	tRF-3
AS-tDR-011395	Asn-GTT-2-3	tRF-3
AS-tDR-011395	Asn-GTT-2-4	tRF-3
AS-tDR-011395	Asn-GTT-2-5	tRF-3
AS-tDR-011395	Asn-GTT-2-6	tRF-3
AS-tDR-011395	Asn-GTT-3-1	tRF-3
AS-tDR-011395	Asn-GTT-3-2	tRF-3
AS-tDR-011395	pre-Asn-GTT-2-1	tRF-1
AS-tDR-011395	pre-Asn-GTT-2-6	tRF-1
AS-tDR-011395	Asn-GTT-1-1	tRF-3
AS-tDR-011395	Asn-GTT-6-1	tRF-3
AS-tDR-011395	pre-Asn-GTT-1-1	tRF-1

### Associated target genes for tRFs

The sequences were loaded to a homemade software based on TargetScan and miRanda, we identified the associated target genes for the three tRFs (AS-tDR-013492, AS-tDR-011395, and AS-tDR-008946). The number of target genes for AS-tDR-013492, AS-tDR-011395, and AS-tDR-008946 is 497, 9114, and 730, respectively.

### Analysis of GO term enrichment

**Fig. 2:**
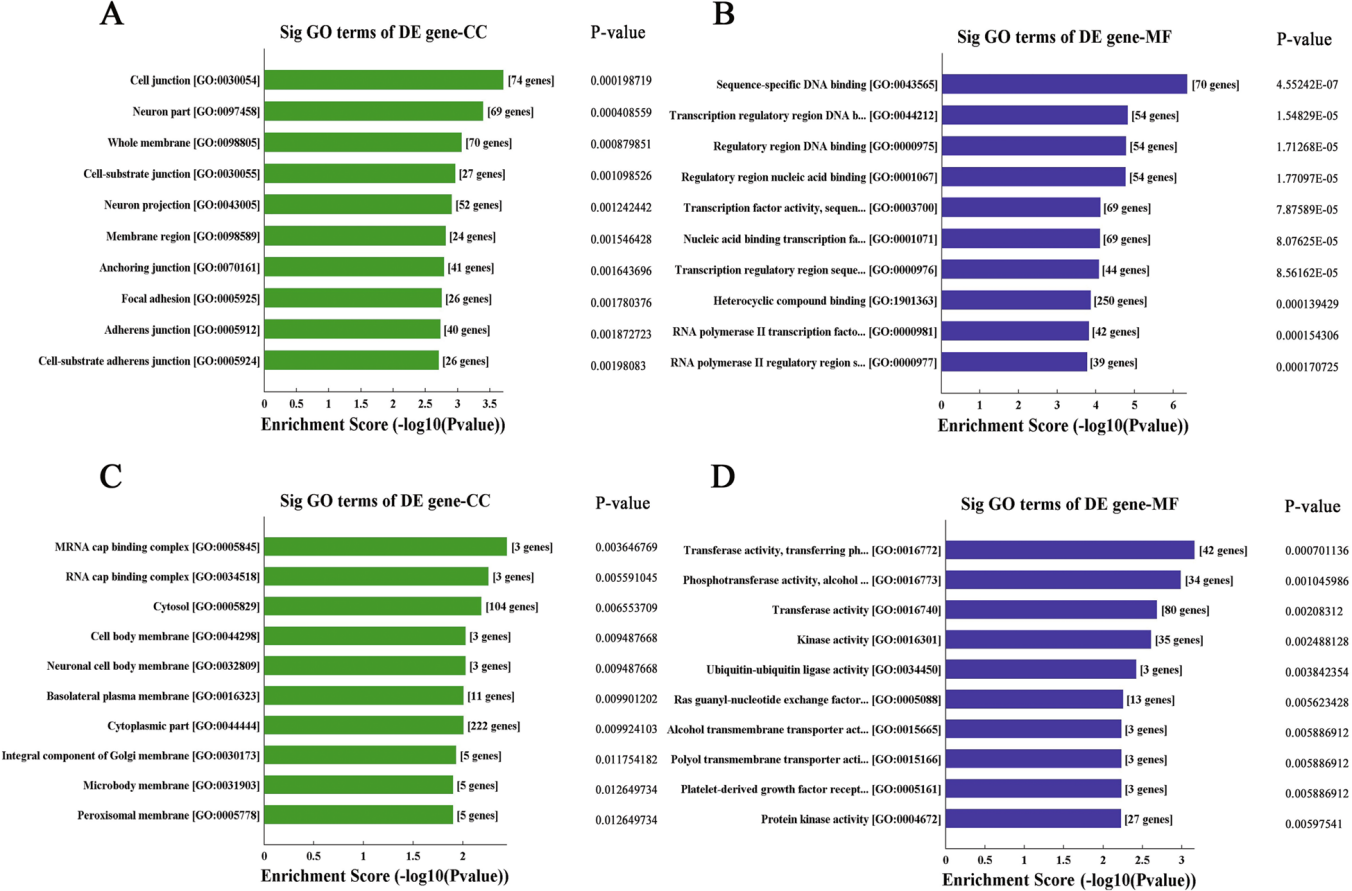
Analysis results of GO term enrichment. GO analysis of the target genes of AS-tDR-008946 was shown in two functional groups: biological processes and cell composition (**a**, **b**). GO analysis of the target genes of AS-tDR-013492 was shown in two functional groups: biological processes and cell composition (**c**, **d**)

Because sequence AS-tDR-011395 has a large number of potential regulatory target genes (9114). It is commonly not reasonable in scientific significance, so we made a functional enrichment analysis for the other two tRFs to get their potential regulatory genes. Biological annotation of the performed using the DAVID online analysis tool, and GO functional enrichment of up-regulated genes were obtained. GO analysis was divided into three functional groups, including molecular function (MF), biological processes (BP), and cell composition (CC). The results are shown in Fig. 2a and b. The results showed that the potential target genes of sequence AS-tDR-008946 tend to be enriched in multiple ways, including cell- substrate adherens junction, focal adhesion, anchoring junction, membrane region, RNA polymerase II regulatory region sequence-specific DNA binding, RNA polymerase II transcription factor activity, transcription regulatory region sequence-specific DNA binding, regulatory region DNA binding, etc. The potential target genes of sequence AS-tDR-013492 tend to be enriched in regulation of response to wounding, rac protein signal transduction, regulation of cellular component biogenesis, regulation of anatomical structure size, actin filament polymerization, immune response-regulating cell surface receptor signaling pathway involved in phagocytosis, regulation of cellular component size, cell body membrane, protein kinase activity, platelet-derived growth factor receptor binding, ras guanyl-nucleotide exchange factor activity, ubiquitin-ubiquitin ligase activity, kinase activity, transferase activity, etc.

### Pathway analysis

**Fig. 3:**
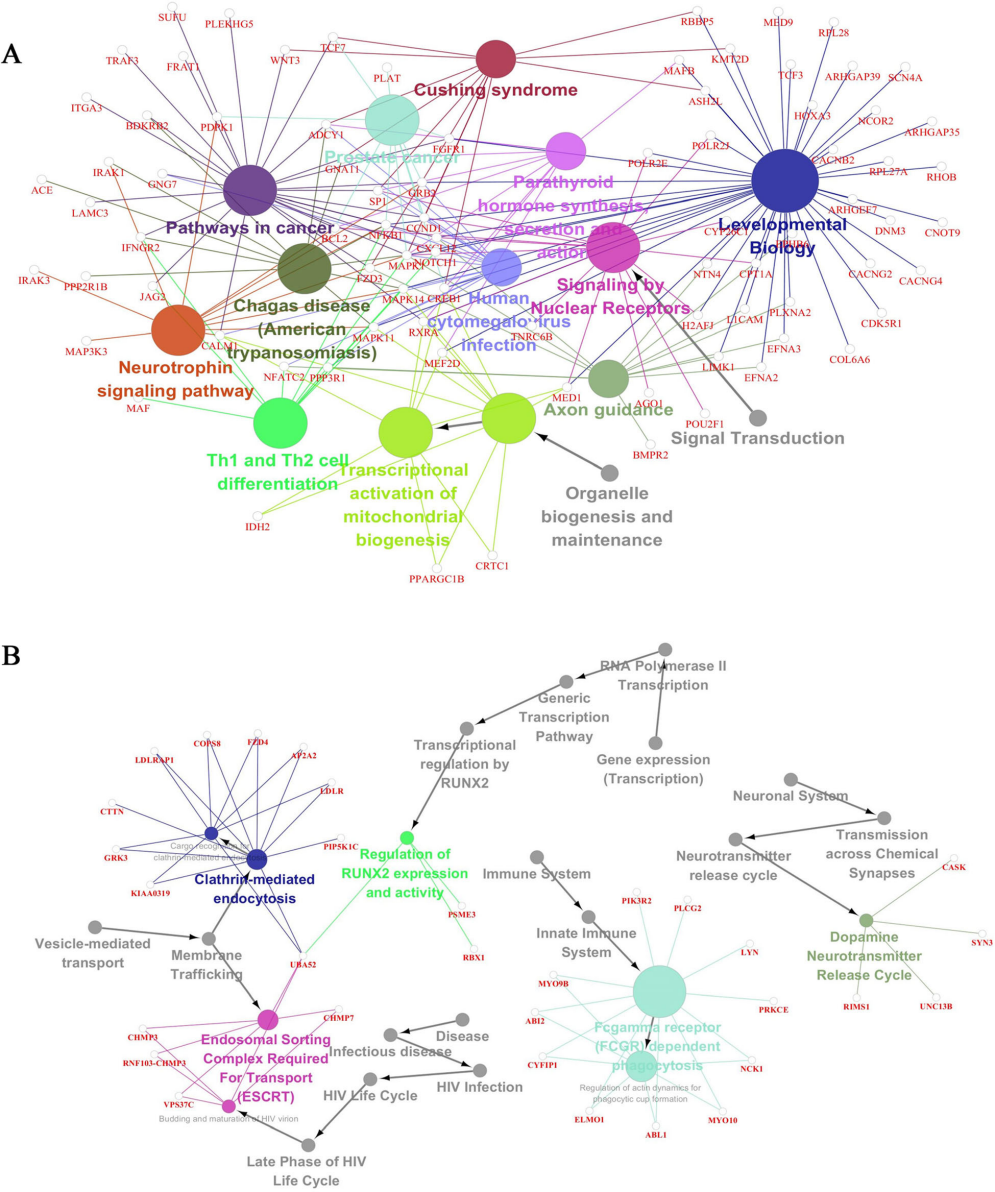
Analysis of the important passways associated with the target genes of AS-tDR-008946 (**a**) and sequence AS-tDR-013492 (**b**). Including Th1 and Th2 cell differentiation, axon guidance, Fcgamma receptor (FCGR) dependent phagocytosis, regulation of actin dynamics for phagocytic cup formation, etc. The size of the node consistent with the number of target genes, the bigger the node is, the more target genes participate in the pathway

Based on the KEGG database, we analyzed the pathways in which the differentially expressed target genes were involved. As shown in Fig. 3, some important pathways targeted by the sequence AS-tDR-008946 and its target genes, including Th1 and Th2 cell differentiation, axon guidance, prostate cancer, developmental biology, human cytomegalovirus infection, pathways in cancer, mitochondrial biogenesis, and transcriptional activation of mitochondrial biogenesis. And important pathways targeted by the sequence AS-tDR-013492 and its target genes including Fcgamma receptor (FCGR) dependent phagocytosis, regulation of actin dynamics for phagocytic cup formation, cargo recognition for clathrin-mediated endocytosis, clathrin-mediated endocytosis, budding and maturation of HIV virion, and Endosomal Sorting Complex Required For Transport (ESCRT).

### Protein-protein interaction (PPI) network and core genes in the PPI network

The PPI network of the target genes number of AS-tDR-008946 comprised of 227 nodes and 480 edges. One hub gene, NEDD4L, was identified by the overlap of the top 20 genes according to four ranked methods in cytoHubba. The PPI network of the target genes number of AS-tDR-013492 comprised of 289 nodes and 572 edges. No hub genes were identified by the overlap of the top 20 genes according to four ranked methods, and a total of seven hub genes were identified by the overlap of the top 20 genes according to three ranked methods, including LYN, FZD4, UBA52, CTTN, ABL1, FGF2, and PDGFB. We also did a preliminary functional validation of UBA52 (a potential target gene of the up-regulated tRF AS-tDR-013492) in several DLBCL cell lines compared with the healthy ones. The result by RT-PCR indicated that, compared with the control, expression of UBA52 was down-regulated significantly in DLBCL cell lines (Additional file 3. Table S1). Our result indicated that UBA52 might be involved in the pathogenesis of DLBCL and AS-tDR-013492 might modulate the expression of UBA52 in a miRNA-like silencing manner. However, the accurate mechanism of this modulation needs further investigation.

## Discussion

In this study, we analyzed the differentially expressed tRF in CD5+ R/R DLBCL patients, identified their potential functions, target genes, and pathways they might be involved in. The results in our study might be beneficial to a better understanding of the mechanisms of pathogenesis and chemotherapy-resistance of CD5+ R/R DLBCL.

Patients with CD5+ DLBCL normally have a worse prognosis, so how to improve their prognosis remains a big challenge for the hematologists. The exact mechanism of the disease progression and chemotherapy-resistance is still uncertain. tRF is a type of highly conserved small non-coding RNAs fragments that are derived from tRNA. Most of the tRF-5 s are present in the nucleus and most of the tRF-3 s and tRF -1 s are cytoplasmic. The characteristics of tRFs such as highly conserved, tissue-specific, time-specific and stably expressing entitled them the qualification to be used as a fluid-based biomarker. This provides a convenient way to study the pathogenesis and development of lymphoma without lymph node/bone marrow biopsy. Studies focus on tRFs and their target genes might be a benefit to exploring novel diagnostic and therapeutic strategies for lymphoma especially more aggressive subtypes.

Till now, functions of tRFs can be deduced by their target genes and pathways they involved in, which were analyzed by bioinformatic tools such as GO and pathway analysis. The subgroup of tRFs can also provide some hints. However, due to the limited quantity of studies about tRFs, not all of the discovered tRFs’ function could be defined. Maybe this deficiency will be improved with the deepening and expansion of research. In this study, we identified 10 tRFs express differently between CD5+ R/R DLBCL patients and the control individuals. We then narrow the study scope to two significantly differently expressed tRF sequences (AS-tDR-008946, AS-tDR-013492) and their target genes were predicted respectively preliminary. The biological functions of the target genes in our study were analyzed through GO annotation in DAVID database (Fig. 2). The majority of the target genes of sequence AS-tDR-008946 were enriched in cell junction items, binding related items, metabolic process, ect. That of sequence AS-tDR-013492 were enriched in the membrane-bounded organelle, cytoplasm, and functions in metabolic processes, cell communication, signal transduction, and protein metabolic process, etc. Plasma membrane and the membrane-bounded structures play important roles in the uptake of drugs and the invasion and metastasis in some malignancy [20, 21]. Previous evidence suggested that ion pumps affect the chemo-resistance of tumor cells and gap junction inhibition can reduce the invasion and metastases of breast cancer and prostate cancer [22–24]. The function of these processes might interpret why tumorous cells in CD5+ DLBCL have a more active metabolic activity and have a close relationship with invasion and chemoresistance. Further analysis of AS-tDR-008946 and AS-tDR-013492 might have a deeper insight into the biological behavior of CD5+ DLBCL cells.

In our study, a good deal of target genes (FGFR1, NEDD4L, RCOR2, and POLR2A) of the differentially expressed tRFs are related to RNA polymerase II. It has been reported that some drug can help to prevent the process of phosphorylation at the serine 2 sites of the C-terminal domain of RNA polymerase II and be used as a potential specific molecular target in NK cell leukemia/lymphoma [25]. Another study suggested that Polymerase II inhibitors may be a useful class of agent for targeting dormant leukemia cells [26]. POLR2A encodes the largest and catalytic subunit of Polymerase II complex and it has been identified to have a long-lasting effect to co-delete with TP53 in human cancers. Inhibiting POLR2A will be a novel therapeutic approach for human cancers [27]. Taken together, based on bioinformatic analysis, the target genes of tRFs we found in our study indicated that the energy metabolism process has been altered by influencing RNA Polymerase II, resulting in the development and progression of the neoplasm. In our study, we also showed that many genes participate in the different pathways in cancer such as TRAF3, which encode a member of the TNF receptor-associated factor (TRAF) protein family and can associate with TNF receptor (TNFR) superfamily which participates in the signal transduction of CD40. Other target genes in our study including ADCY1, BCL2, BDKRB2, CALM1, and CCND1 have been proved to play important roles in multiple pathways of cancer progression. We surmise that a series of genetic changes occurred in CD5+ R/R DLBCL cells and related pathways which may lead to the resistance of chemotherapeutic drugs through different mechanisms including affecting the metabolic process of drugs, cell adhesion, binding, and apoptosis.

In the current study, target genes of AS-tDR-008946 and sequence AS-tDR-013492 participate in Th1 and Th2 cell differentiation (Fig. 3). Mori observed that T-helper (Th)1/Th2 imbalance happen in different kinds of pathological conditions such as DLBCL. In patients in CR, the Th1/Th2 balance was polarized to Th1 and this result showed that a Th1/Th2 imbalance could affect greatly in the lymphomagenesis and durable remission of DLBCL [28]. In the other two studies, the Th1/Th2 balance was proved could be used for evaluating prognosis [29, 30]. It is obvious that Th1 and Th2 cell differentiation is an important part of our study and cannot be ignored. T cell-mediated immunity affects the occurrence and development of DLBCL in many ways such as enhancing antitumor response. After receiving chemotherapy, the balance between Th1 and Th2 cells may be broken and the changes can associate with the different responses to treatment.

**Fig. 4:**
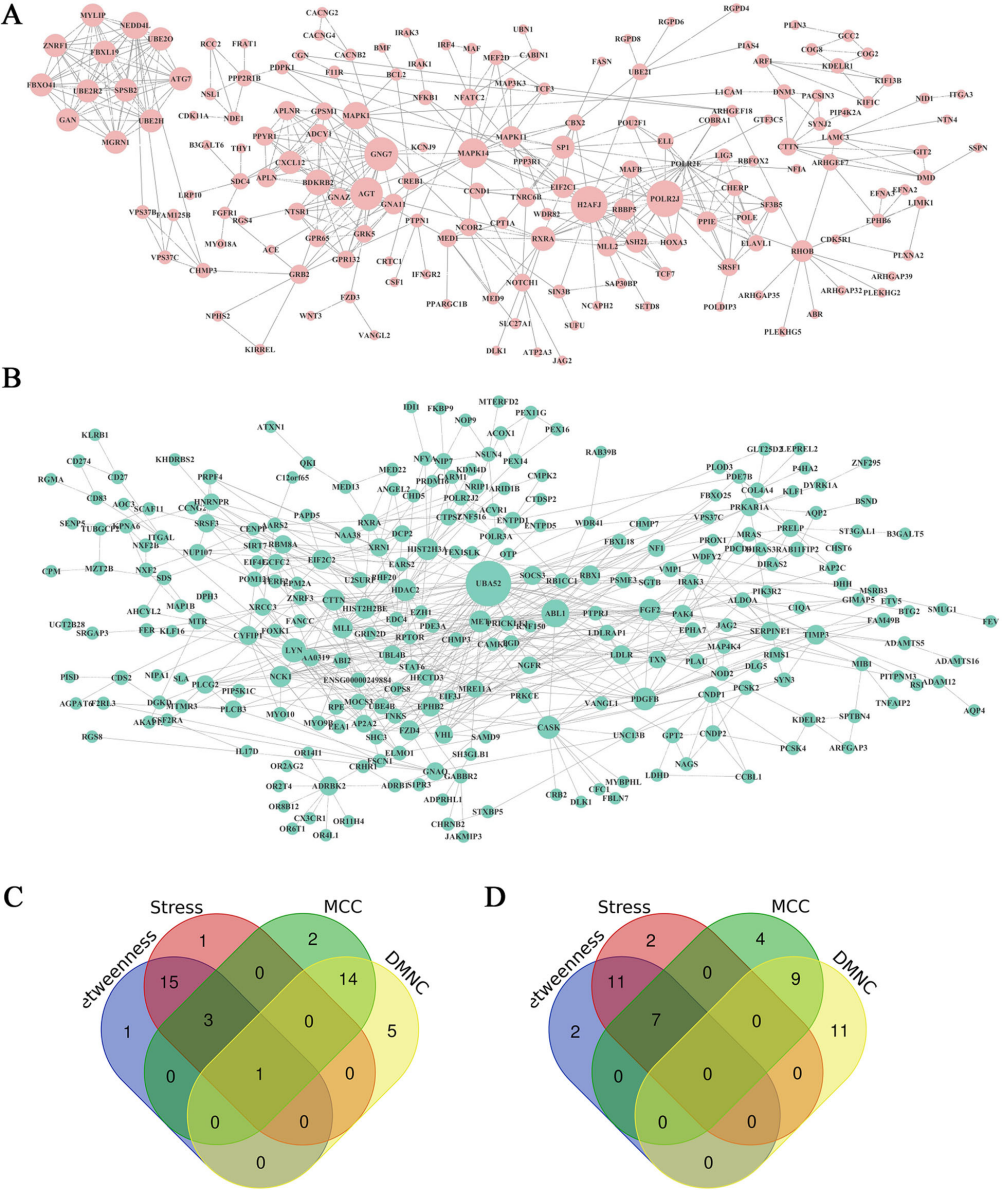
Protein-protein interaction network and core genes in the PPI network of AS-tDR-008946 (**a**) and sequence AS-tDR-013492 (**b**). The size of the Nodes coincides with the degree of the gene. Bigger size indicates that the node has a higher degree. Venn diagram of the top 20 hub genes of AS-tDR-008946 (**c**) and sequence AS-tDR-013492 (**d**) in betweenness, stress, MCC and DMNC

Through PPI network construction, a series of hub proteins have been observed to form a local network, including NEDD4L and UBA52, *etc* (Fig. 4). Many of them were reported associated with the development and progression of different kind of cancers and drug resistance, including lymphoma [31–33]. NEDD4L (Neural Precursor Cell Expressed, Developmentally Down-Regulated 4-Like, E3 Ubiquitin Protein Ligase) is a protein-coding gene and the downregulation of it has been proved related to poor prognosis in hepatocellular, ovarian epithelial cancer, lung cancer [34–36]. It is necessary for the maintenance of the PI3K/AKT signaling pathway and can negatively regulate PIK3CA protein levels via ubiquitination. An increasing number of researches indicated that NEDD4L was related to some tumor progression pathways and was found abnormally expressed in several kinds of solid cancers [37, 38]. The downregulation of NEDD4L can also promote tumor growth and inhibit the MAPK/ERK signal pathway [36]. Both PI3K/AKT and MAPK/ERK pathway play an important role in the pathogenesis of B cell lymphoma. In our study, both AS-tDR-008946 and AS-tDR-013492 are upregulated and it may inhibit the expression of NEDD4 through a way similar to miRNA and thus accelerate the progress of lymphoma. So NEDD4 might be an important entry point to study chemoresistance of CD5+ R/R DLBCL. Another hub protein named UBA52 which is fused to the ribosomal proteins L40 (RPL40) can encode ubiquitin in normal cell process. The ubiquitin system regulates almost all aspects of cellular function and has massive, multilevel influences in the control of the cell cycle progression and DNA damage responses [39]. It can also play important roles in tumorigenesis, for example, UBA52 is overexpressed in colon cancer and renal cancer [40, 41]. It also has been proved that the LUBAC ubiquitin ligase can be an important therapeutic target in DLBCL [42]. Based on our findings, the predicted hub proteins by informatic analysis including NEDD4L and UBA52 provide us valuable clue to investigate the mechanism of tumorigenesis. In turn, the upregulation of AS-tDR-008946 and AS-tDR-013492 might influence the gene expressions by modulating important pathways (PI3K/AKT or MAPK/ERK) or cellular processes such as ubiquitin. From another perspective, agents targeting PI3K/AKT and MAPK/ERK pathways might benefit the prognosis of CD5+ R/R DLBCL.

However, what can we do to utilize tRF as the bio-target to inhibit the lymphoma cell proliferation? Former studies have confirmed that knockdown of an overexpressed tRF can inhibit cancer cell proliferation and the recovery of lacking tRF can prevent cancer cell metastasis [43]. In human embryonic kidney cells 293(HEK293), tRFs are associated with Argonautes 1, 3, and 4, and have very similar properties to miRNAs, indicating that tRFs may play an important role in RNA silencing [44]. It has also been proved that the level of tRF could affect the efficacy of miRNAs and siRNAs [44]. Taken together, the upregulation of the two tRFs sequences (AS-tDR-008946, AS-tDR-013492) in our study might promote the CD5+ DLBCL progression through silencing of related genes, have the functions similar to some microRNAs or affecting the efficacy of miRNAs and siRNAs. New drugs targeting these tRFs might be a promising strategy to improve the prognosis of CD5+ R/R DLBCL.

## Conclusions

In this study, we identified differentially expressed tRFs profiles in patients with CD5+ R/R DLBCL and validate our sequencing results by qRT-PCR. We also analyzed the functions of the target genes with multiple bioinformatic tools. PI3K/AKT and MAPK/ERK are the most frequently influenced pathways by tRFs associated genes. As a result, two important hub genes including NEDD4L and UBA52 were identified and they might play crucial roles in signal transmission and RNA synthesis, respectively. New drugs targeting them might benefit the therapeutic effect of CD5+ DLBCL. Although the sample size in our study is a bit of small, the preliminary results we got here might provide a valuable indication for better understanding the pathogenesis and progression of CD5+ R/R DLBCL. In the following study, we will expand the sample size and carry out the functional investigation for further verification. Future study on these tRFs and associated hub genes will provide more laboratory evidence and develop novel treatment strategies for CD5+ R/R DLBCL.

## Reviewers’ comments

### Reviewer’s report 1

Zhen Qing Ye

**Reviewer summary**

The authors have improved the manuscript greatly in this revision version, even for some points I’m not convinced yet by their response. The main concern from me is the reliability of of results as bio-markers, because so less number of samples and those samples were not from tumor cells but peripheral venous blood samples. All these factors will dramatically reduce the reliability due to the large statistical fluctuation. But this can be viewed as a scientific controversy, rather than as the reason for accepting/reject on the manuscript. They have addressed many other concerns I have raised previously, but the figure quality looks still not good, at least in the PDF version. For example, in Fig. 1a and b, it is still very hard to read those text labels.

Authors’ response: *We appreciate the reviewer’s professional comments and kind help during our revision. As we have mentioned in our former response letter, this study is a preliminary exploration of the correlation between tRFs and CD5+ R/R DLBCL. Results from the bioinformatic analysis provided some research clues for further study, so we will expand our sample size and do more validation to confirm our results. We will timely summary the results and submit our new data to the journal*.

*We have enlarged the size and the pixel of each figure. Currently, each image size is about 70–90 M. We converted the .jpg format to .eps format and uploaded them separately. We hope they can meet the quality criteria of the journal for publication. Furthermore, the reviewer can check the figures through the link provided in the pdf version.*

### Reviewer’s report 3: Jin Zhuang Dou

**Reviewer comments to Authors**

1. In my previous comment 2), I hope the authors can explain why there are 406 tRFS&tiRNA specific to the control. I would like to see some evidence to support these tRFS&tiRNA, otherwise, it is hard to convince me about the downstream analysis.

Authors’ response: *We are very sorry that we have not explained this question in our former revision clearly. And we tried to reinterpret it as follows:*

*In the progress of cell division, unexpected somatic mutations are common and most of them can be modified by the normal genetic repair mechanism. Former studies have confirmed that small non-coding RNAs play important roles in the cellular and tissue homeostasis by RNA-mediated gene silencing mechanism. As a subtype of non-coding RNA, tRFs play roles similar to microRNAs in some biological processes. Different from microRNAs, tRFs are highly conserved, tissue-specific and time-specific and are reported to express stability in almost all types of cells and organisms and can be detected in body fluid. They are found to exist widely in the eukaryotic biological process. In this study, Illumina NextSeq analysis revealed 406 tRFs&tiRNAs specific to the control. We deduce that in the control group, they might function in the maintenance of normal cellular physiology, or in some content, they might participate the tumorigenesis inhibition. Fow now, a tremendous amount of work is still necessary to verify their accurate function in the normal individual.*

In the present study, the tRFs specific to the patients with CD5+ R/R DLBCL indicated that they might have a close attachment to the lymphoma development and progression. On the other hand, 406 tRFs&tiRNAs specific to the control might be the inhibiting factors of lymphoma. However, they might also participate in the inhibition of other diseases. Therefore, we are not sure whether these 406 tRFs&tiRNAs are all definitely associated with current disease status in our study. It is difficult to draw a more accurate conclusion due to the limited amount of literature associated with tRFs. In the future, more illustration of their function might be generated from further studies.

Combining varies experimental techniques to determine the further research target can remedy the shortage of a single method and improve the reliability of research. In our study, we tried to analyze four fragments (AS-tDR-011383, AS-tDR-013475, AS-tDR-013487, AS-tDR-011395) among the 406 tRFs&tiRNAs according to the fold change and *p*-value. However, 3 three of them were excluded because there are no significant differences between the experimental group and the control group by RT-PCR validation. AS-tDR-011395 was also excluded for further analysis because subsequent bioinformatic analysis indicated too many (9114) target genes.

To further explain the functions of 406 tRFs&tiRNAs, we performed the classification according to their cleavage position in tRNA and the result is shown in Additional file 1: Figure S1B. Until now, tRFs can be divided into tRF-1, tRF-2, tRF-3, tRF-5, and i-tRF. Some articles showed that tRF-3 s and tRF-5 s can behave similarly to miRNAs in human cell lines and interact with Argonaute proteins. Through Argonaute co-operation, tRFs can impact a number of cellular functions such as proliferation and mediating RNA inactivation. For example, it was demonstrated that a tRF-3 from tRNA-Gly-GCC is abundantly expressed in naïve, germinal center, and memory B cells in humans and is physically associated with Ago proteins [11]. It has also been confirmed that this tRF-3 was not expressed in transformed B cell or lymphoma biopsies, suggesting that this tRF is specific to the normal cells. Due to the word count limit, the above explanation has not been added to the manuscript. We uploaded the figure and the corresponding detailed explanation as Additional file 1: Figure S1.

2. In my previous comment 3), I hope the authors can provide some biological explanations about the 10 clusters. I agree that cluster diagrams indicate the expression patterns between samples or genes. Unfortunately, no functional insights are obtained here from this heatmap.

Authors’ response: *In the present study, we used K-means clustering algorithm to separated the differentially expressed* tRFs&tiRNAs *between the test & control group. We uploaded the raw data for heatmap as the* Additional file 2* in the revised manuscript. As shown in the heatmap* (Fig. 1b), *each row represents one gene and all selected genes were categorized into 10 clusters according to their expression level based on K-means clustering. Each column represents one sample. Blue represents an expression level below the mean and red represents an expression level above the mean. After clustering, the expression similarity of the* tRFs&tiRNAs *in the same cluster is as large as possible, and the expression difference of* them *which are not in the same cluster is also as large as possible. That is the most ideal results for clustering. Therefore,* Fig. 1b *also showed a distinguishable tRF & tiRNA expression profiling among samples. In other words, genes in the same cluster might have different functions, this might be the reason why we can not figure out their functions among clusters. We do agree with the reviewer’s suggestion, functional classification is much important to illustrate the roles of associated* tRFs&tiRNAs in the disease group. So we determined several sequences according to fold change and *p*-value as well as the result of RT-PCR and performed corresponding functional analyzations through *target gene prediction, GO term enrichment analyzation, pathway analyzation, and protein-protein interaction (PPI) network analyzation. Please refer to Page 9-Page 11.*

## Supplementary information

**Additional file 1: Figure S1.** (A) The classification of 308 tRFs&tiRNAs specific to patients with CD5+ R/R DLBCL according to their cleavage position in tRNA. (B) The classification of 406 tRFs&tiRNAs specific to control according to their cleavage position in tRNA. Due to the limited amount of literature associated with tRFs, it is difficult to draw accurate conclusions about the functions of these fragments. Here we take an example from literature to illustrate their potential function. It was reported that a tRF-3 from tRNA-Gly-GCC is abundantly expressed in naïve, germinal center, and memory B cells in humans and is physically associated with Ago proteins. It has also been confirmed that this tRF-3 was not expressed in transformed B cell or lymphoma biopsies, suggesting that this tRF is specific to the normal cells. **Figure S2.** (A) The Scatter plots of tRFs & tiRNAs between two groups. TPM values of all tRFs & tiRNAs are plotted. The values of X and Y axes in the Scatter-Plot are the averaged TPM values of each group (log2 scaled). Genes above the top line (red dots, up-regulation) or below the bottom line (green dots, down-regulation) indicate more than 2.0 fold change (Default fold change value is 2.0) between two compared groups. Gray dots indicate tRFs & tiRNAs without differential expression. (B) The Volcano Plots of tRFs & tiRNAs for Test vs Control. Red/Green circles indicate 2.0 fold change differentially expressed tRFs & tiRNAs with statistical significance (Red: up-regulated; Green: down-regulated). Gray circles indicate non-differentially expressed tRFs & tiRNAs, whether FC or q-value is not satisfied. The values of X and Y axes in the Volcano Plot are the Fold change (log2 transformed) and *p*-value (−log10 transformed) between two groups, respectively. (C) Examples of tRNA structure for each fragment (AS-tDR-013492 and AS-tDR-008946). The sequence of AS-tDR-013492 comes from two different tRNAs, and we depicted the location of the fragments with red lines. **Figure S3.** In DLBCL cell lines, UBA52 expression is significantly down-regulated.

**Additional file 2.** Raw data of heatmap (Figure 1B).

**Additional file 3.** **Table S1.** The preliminary RT-PCR result of UBA52 in several DLBCL cell lines compared with the healthy ones.

## Data Availability

Please contact the author for data requests.
