# Supplementary material for: Differentially expressed tRFs in CD5 positive relapsed & refractory diffuse large B cell lymphoma and the bioinformatic analysis for their potential clinical use
Source: Biol Direct. 2019 Nov 27;14:23. doi: 10.1186/s13062-019-0255-8 (PMC6882323; doi:10.1186/s13062-019-0255-8)
Supplement: Supplementary file 1 — Additional file 1: Figure S1. (A) The classification of 308 tRFs&tiRNAs specific to patients with CD5+ R/R DLBCL according to their cleavage position in tRNA. (B) The classification of 406 tRFs&tiRNAs specific to control according to their cleavage position in tRNA. Due to the limited amount of literature associated with tRFs, it is difficult to draw accurate conclusions about the functions of these fragments. Here we take an example from literature to illustrate their potential function. It was reported that a tRF-3 from tRNA-Gly-GCC is abundantly expressed in naïve, germinal center, and memory B cells in humans and is physically associated with Ago proteins. It has also been confirmed that this tRF-3 was not expressed in transformed B cell or lymphoma biopsies, suggesting that this tRF is specific to the normal cells. Figure S2. (A) The Scatter plots of tRFs & tiRNAs between two groups. TPM values of all tRFs & tiRNAs are plotted. The values of X and Y axes in the Scatter-Plot are the averaged TPM values of each group (log2 scaled). Genes above the top line (red dots, up-regulation) or below the bottom line (green dots, down-regulation) indicate more than 2.0 fold change (Default fold change value is 2.0) between two compared groups. Gray dots indicate tRFs & tiRNAs without differential expression. (B) The Volcano Plots of tRFs & tiRNAs for Test vs Control. Red/Green circles indicate 2.0 fold change differentially expressed tRFs & tiRNAs with statistical significance (Red: up-regulated; Green: down-regulated). Gray circles indicate non-differentially expressed tRFs & tiRNAs, whether FC or q-value is not satisfied. The values of X and Y axes in the Volcano Plot are the Fold change (log2 transformed) and p-value (−log10 transformed) between two groups, respectively. (C) Examples of tRNA structure for each fragment (AS-tDR-013492 and AS-tDR-008946). The sequence of AS-tDR-013492 comes from two different tRNAs, and we depicted the location of the fragments with red li [file 13062_2019_255_MOESM1_ESM.docx]

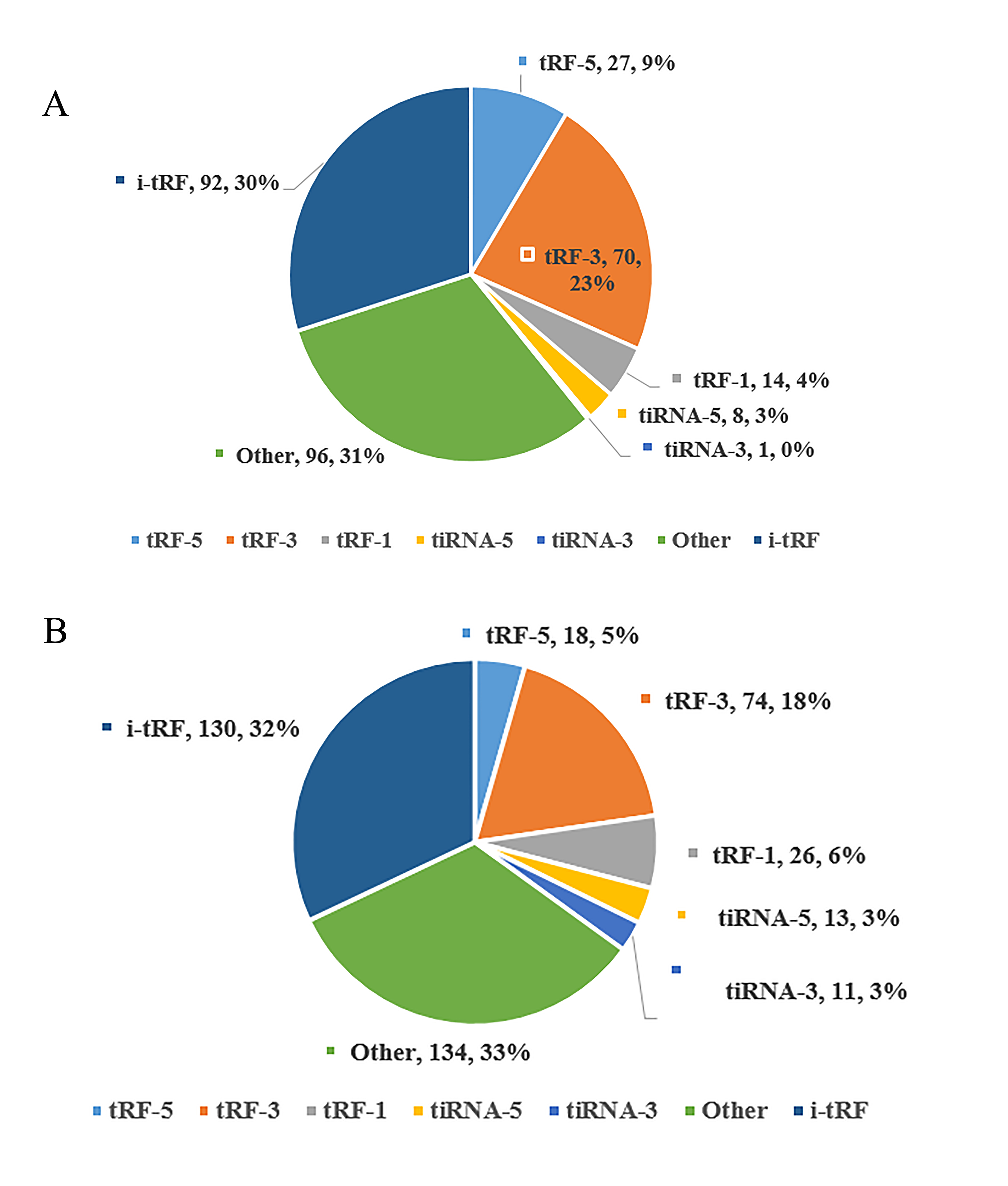


**Figure S1**. (**A**)The classification of 308 tRFs&tiRNAs specific to patients with CD5+ R/R DLBCL according to their cleavage position in tRNA. (**B**)The classification of 406 tRFs&tiRNAs specific to control according to their cleavage position in tRNA. Due to the limited amount of literature associated with tRFs, it is difficult to draw accurate conclusions about the functions of these fragments. Here we take an example from literature to illustrate their potential function. It was reported that a tRF-3 from tRNA-Gly-GCC is abundantly expressed in naïve, germinal center, and memory B cells in humans and is physically associated with Ago proteins. It has also been confirmed that this tRF-3 was not expressed in transformed B cell or lymphoma biopsies, suggesting that this tRF is specific to the normal cells.


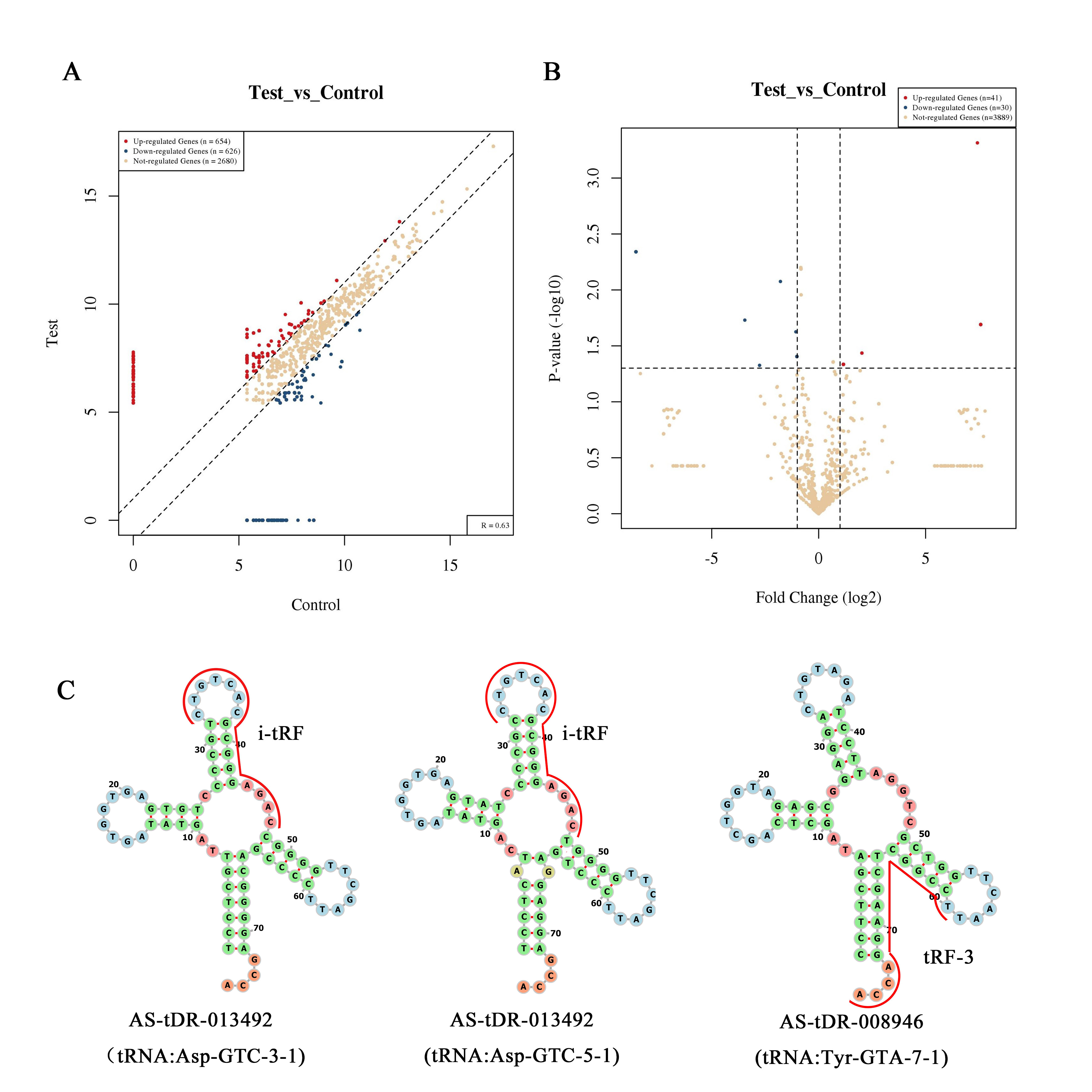


**Figure S2**. （**A**）The Scatter plots of tRFs & tiRNAs between two groups. TPM values of all tRFs & tiRNAs are plotted. The values of X and Y axes in the Scatter-Plot are the averaged TPM values of each group (log2 scaled). Genes above the top line (red dots, up-regulation) or below the bottom line (green dots, down-regulation) indicate more than 2.0 fold change (Default fold change value is 2.0) between two compared groups. Gray dots indicate tRFs & tiRNAs without differential expression. (**B**) The Volcano Plots of tRFs & tiRNAs for Test vs Control. Red/Green circles indicate 2.0 fold change differentially expressed tRFs & tiRNAs with statistical significance (Red: up-regulated; Green: down-regulated). Gray circles indicate non-differentially expressed tRFs & tiRNAs, whether FC or q-value is not satisfied. The values of X and Y axes in the Volcano Plot are the Fold change (log2 transformed) and p-value (-log10 transformed) between two groups, respectively. (**C**) Examples of tRNA structure for each fragment (AS-tDR-013492 and AS-tDR-008946). The sequence of AS-tDR-013492 comes from two different tRNAs, and we depicted the location of the fragments with red lines.

**Figure S3**. In DLBCL cell lines, UBA52 expression is significantly down-regulated.
